# Advanced imaging for the diagnosis of age‐related macular degeneration: a case vignettes study

**DOI:** 10.1111/cxo.12607

**Published:** 2017-10-09

**Authors:** Angelica Ly, Lisa Nivison‐Smith, Barbara Zangerl, Nagi Assaad, Michael Kalloniatis

**Affiliations:** ^1^ Centre for Eye Health Sydney New South Wales Australia; ^2^ School of Optometry and Vision Science The University of New South Wales Sydney New South Wales Australia; ^3^ Department of Ophthalmology Prince of Wales Hospital Sydney New South Wales Australia

**Keywords:** age‐related macular degeneration, case vignettes, diagnosis, imaging, optometrist

## Abstract

**Background:**

The aim of this study is to evaluate the diagnosis, staging, imaging and management preferences, and the effect of advanced imaging among practising optometrists in age‐related macular degeneration (AMD).

**Methods:**

Up to 20 case vignettes (computer‐based case simulations) were completed online in a computer laboratory in random order by 81 practising optometrists of Australia. Each case presented findings from a randomly selected patient seen previously at the Centre for Eye Health for a macular assessment in the following order: case history, preliminary tests and colour fundus photography. Participants were prompted to provide their diagnosis, management and imaging preference. One additional imaging result (either modified fundus photographs and infrared images, fundus autofluorescence, or optical coherence tomography [OCT]) was then provided and the questions repeated. Finally, all imaging results were provided and the questions repeated a third time.

**Results:**

A total of 1,436 responses were analysed. The presence of macular pathology in AMD was accurately detected in 94 per cent of instances. The overall diagnostic accuracy of AMD was 61 per cent using colour fundus photography. This improved by one per cent using one additional imaging modality and a further four per cent using all imaging. Across all responses, a greater improvement in the diagnostic accuracy of AMD occurred following the presentation of OCT findings (versus other modalities). OCT was the most preferred imaging modality for AMD, while multimodal imaging was of greatest benefit in cases more often misdiagnosed using colour fundus photography alone. Overall, the cohort also displayed a tendency to underestimate disease severity.

**Conclusion:**

Despite reports that imaging technologies improve the stratification of AMD, our findings suggest that this effect may be small when applied among practising optometrists without additional or specific training.

Age‐related macular degeneration (AMD) is a leading cause of visual impairment worldwide, projected to affect 196 million people by the year 2020.^1^ There is a growing body of evidence showing that patients (especially with the neovascular form) benefit from early detection.^2^, ^3^ Thus, primary eye‐care providers are mandated to provide early and accurate diagnosis of AMD and to instigate appropriate management, including advice about smoking cessation, dietary changes or supplementation, Amsler grid self‐monitoring and an ongoing care plan.^4^, ^5^ Staging of the disease is also important: early and intermediate stages may be detectable before the onset of symptoms and the stage of disease should be used to guide the management plan. For instance, patients with intermediate AMD may be recommended nutritional supplements, while cases with advanced neovascular AMD should be referred promptly to an ophthalmologist for treatment.^2^

There has been a paradigm shift toward the integration of ocular imaging technologies (optical coherence tomography [OCT], fundus autofluorescence [FAF] and near infrared reflectance imaging) into the routine clinical assessment of AMD cases by eye‐care professionals.^2^, ^6^, ^7^ Although current classification schemes and grading scales rely primarily on retinal photography,^8^, ^9^ imaging technologies are designed to facilitate diagnosis and risk‐related staging, and have been associated with high sensitivity.^10^, ^11^, ^12^, ^13^, ^14^, ^15^, ^16^, ^17^, ^18^, ^19^, ^20^ They may also be mandatory for the diagnosis of AMD subtypes, such as polypoidal choroidal vasculopathy.

Despite advances in evidence‐based practice and imaging, AMD cases may still be misdiagnosed or not appropriately managed^21^, ^22^, ^23^ as practising clinicians may not receive any formal training or accreditation on the interpretation of imaging and may be unsure of how to interpret the results.^24^, ^25^ There is also a paucity of data relating to the use of these devices and variation in clinical eye‐care by practising optometrists. Such practice variation may be undesirable. For example, in neovascular AMD, inappropriate management variation has the potential to contribute to preventable vision loss. Finally, the impact of imaging on patient management is not clear.

Thus, the primary aim of this study was to evaluate the staging, diagnosis, imaging and management preferences and the effect of advanced imaging among practising optometrists in AMD using a series of case vignettes (computer‐based simulation of patient cases). This could enhance our understanding of the use of imaging in AMD by optometrists.

## METHODS

### Study design and setting

Participating optometrists were recruited using the mailing list of the Centre for Eye Health (CFEH; an establishment that provides imaging and visual system diagnostic services to the general community). Data were collected using a series of case vignettes administered online using Survey Monkey (Survey Monkey Inc., https://www.surveymonkey.com/) and Articulate Online (Articulate Global Inc., https://en-au.articulate.com/). Participants were invited to complete the cases during one whole‐day continuing professional development event, hosted by the same organisation, on topics unrelated to AMD. Each participant was seated for three 45‐minute sessions in a computer room using a pre‐calibrated monitor to complete a maximum of 20 case vignettes (40 eyes; 10 AMD cases and 10 non‐AMD cases combining a mixture of normal and diseased maculas) presented in random order.

Before the case vignettes, participants were briefed about the study purpose. Consistent with published recommendations relating to the conduct of case vignettes,^26^ participants were unambiguously advised to respond as they would to a survey, that is, as they would do in clinical practice. They were explicitly advised that the survey would showcase high‐resolution images from a series of 20 patients that may have AMD (Figure S1). The responses would be confidential and analysed on a group level only (rather than on an individual level) and these points were regularly reiterated throughout the event. Participants were provided with a brief, online entering questionnaire (abridged from a previous study) in order to ascertain basic demographic, experience and professional characteristics. All study participants and patients (from which the case images were acquired) provided written consent in accordance with the Declaration of Helsinki, approved by a Biomedical Human Research Ethics Advisory Panel of the University of New South Wales, Sydney, New South Wales, Australia.

### AMD case vignettes

**Figure 1:**
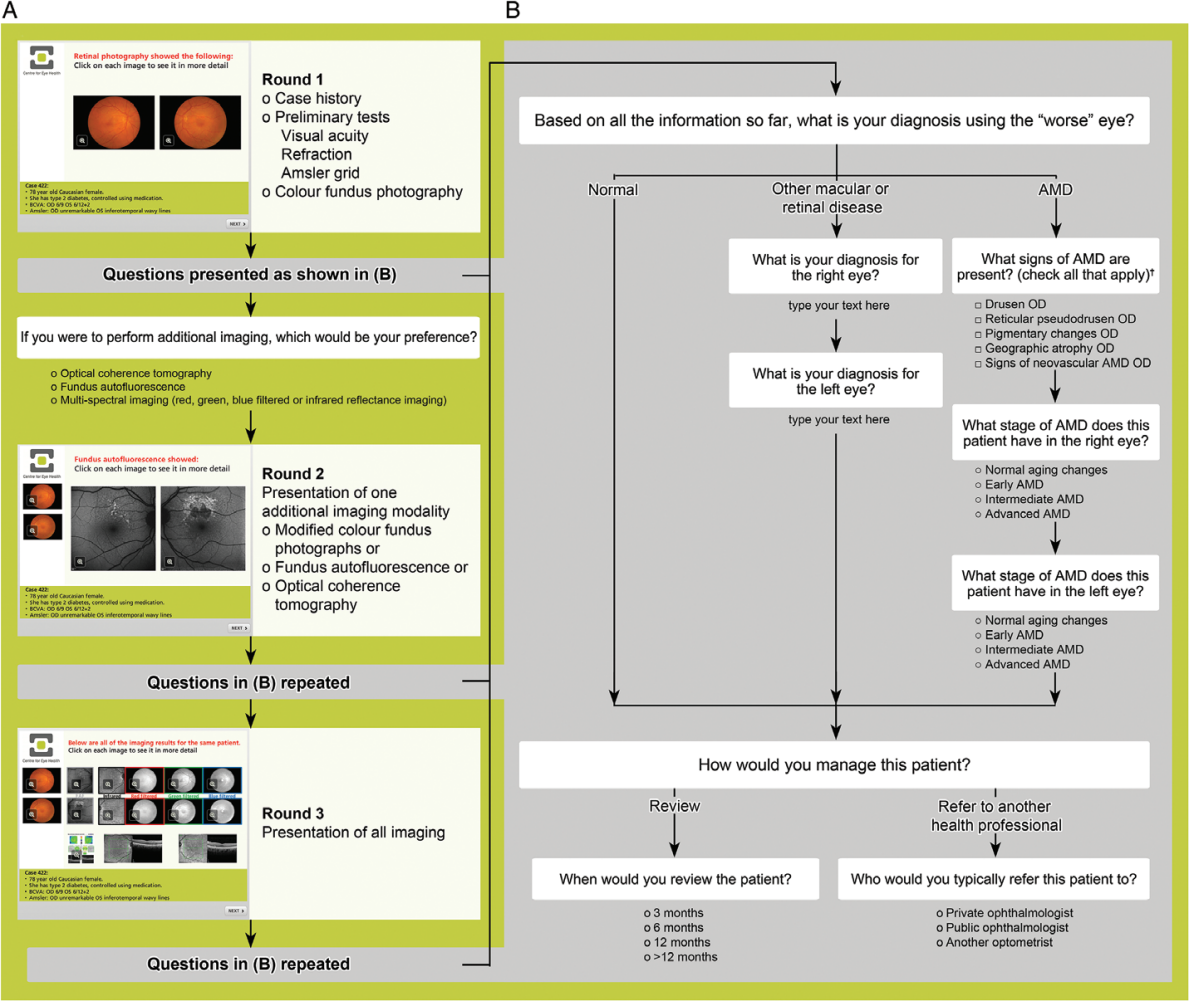
A: Concept flowchart illustrating the questions and data captured in each case vignette (computer‐based case simulation). Each case is presented as a clinical scenario in a slide‐show format whereby the participant first reads about the patient's case history and preliminary test findings (visual acuity, refraction and Amsler grid). Colour fundus photographs are then presented and the participant queried regarding their diagnosis, management and imaging preferences (round one). B: The questions (other than imaging preference) are repeated following presentation of images using one imaging modality (round two), and then again following presentation of all imaging results (round three). Participants had the option of reviewing the fundus photographs at any time. ^†^Response options for the left eye were also provided. AMD: age‐related macular degeneration, OD: right eye.

Each case vignette presented ocular findings from both eyes using a slide‐show format in a sequence analogous to the order typically encountered in clinical practice: case history, preliminary testing (visual acuity, refraction and Amsler grid findings) and non‐stereoscopic, mydriatic colour fundus photography. Following the presentation of fundus photographs, participants were prompted to answer four to seven questions indicating their diagnosis (from three options: normal, other macular or retinal disease or AMD), the signs and stage of AMD present (in instances where AMD was selected as the diagnosis), management plan and imaging preference. Questions were a combination of multiple choice and open‐ended free response, including two filter questions (Figure 1).

Participants were then shown one additional, prospectively randomised, imaging result (either modified ‘filtered’ fundus photographs and an infrared image, FAF or OCT) and the questions were repeated. Finally, they were provided with all of the imaging results (that is, modified fundus photographs and infrared imaging, FAF and OCT) and the queries were repeated a third time. Participants were not allowed to modify submitted answers at any stage of the case vignette.

Each case vignette was revised and pilot‐tested with 10 CFEH optometrists for clarity and content validity. Minor modifications to the format were made and extraneous information removed. Pilot data were not included in the final analysis. Based on the pilot data, a minimum sample size of 67 participants was required in order to demonstrate a statistically significant difference in proportions with 80 per cent power at a 95 per cent confidence level (assuming a 0.4 moderate correlation between paired observations).^27^ Ten cases with varying macular changes related to AMD were included based on the assumption of a 10 per cent false positive rate.

### Case images

**Figure 2:**
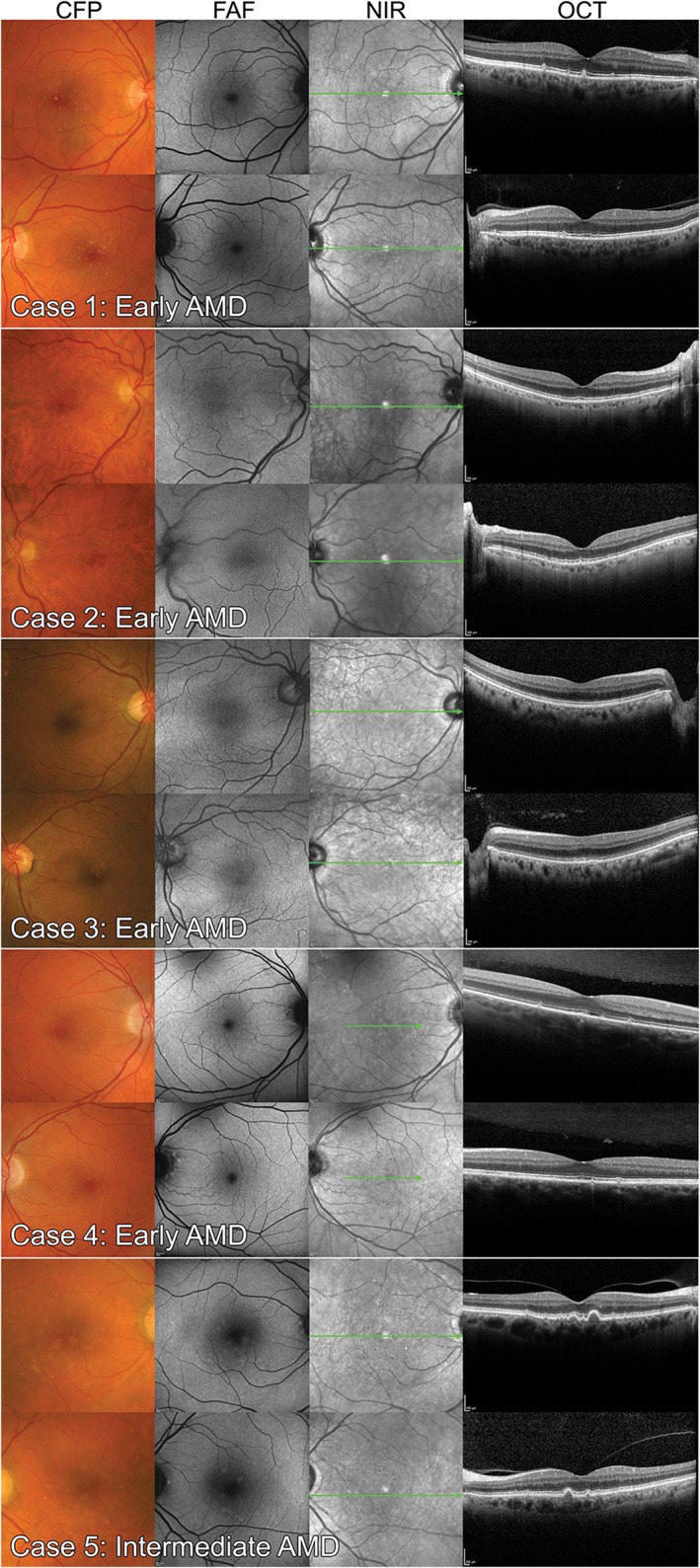
Case images from four persons with early age‐related macular degeneration (AMD) and one person with intermediate AMD used in the study. The Beckman classification defined early AMD in persons with medium drusen only (between 63 and 125 μm in diameter). Intermediate AMD describes persons with large drusen (≥125 μm) or pigmentary changes associated with at least medium drusen. Case labels are consistent with Figure 7 and Tables S1 and S3. CFP: colour fundus photography, FAF: fundus autofluorescence, NIR: near infrared reflectance, OCT: optical coherence tomography.

**Figure 3:**
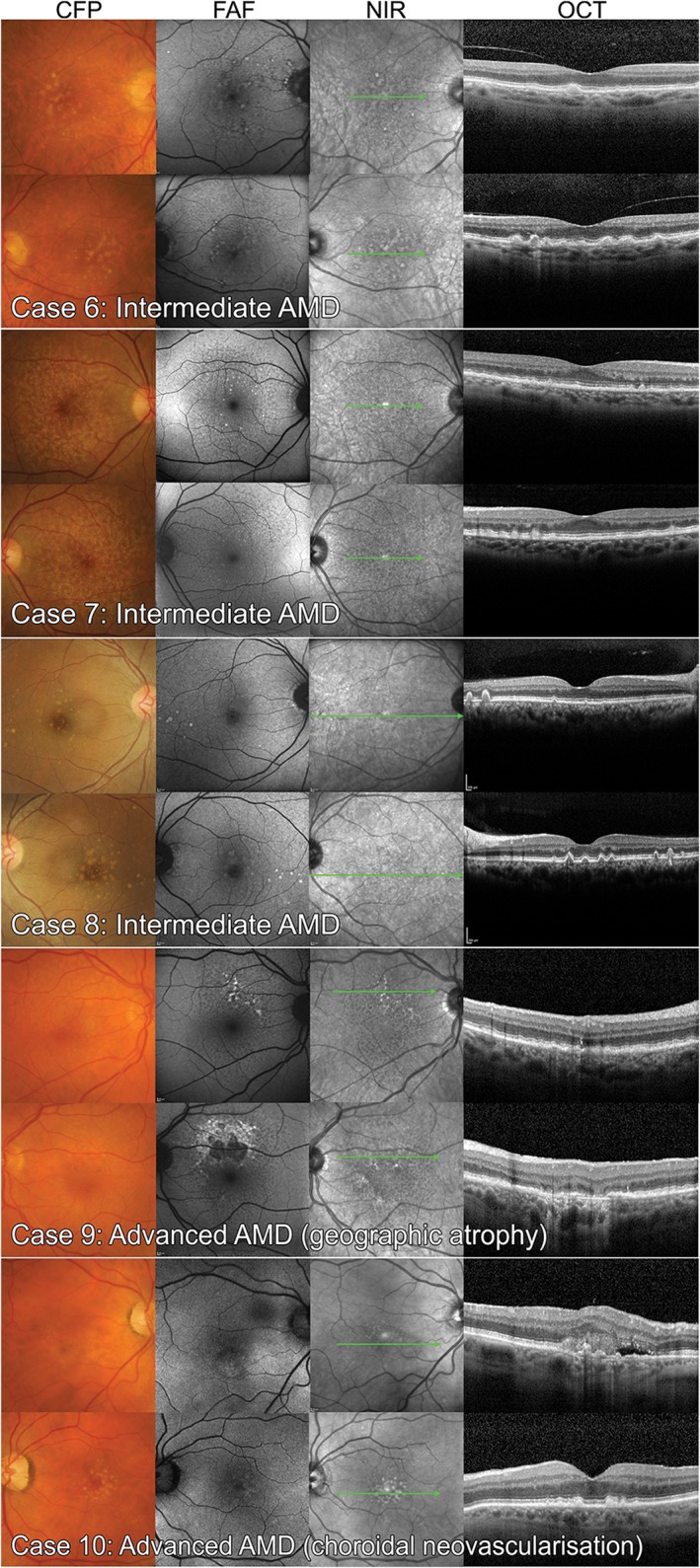
Case images of six eyes (three bilateral cases) with intermediate age‐related macular degeneration (AMD) and two cases with advanced AMD featured in the study; the left eye of case 9 has foveal, multilobular geographic atrophy while the right eye of case 10 has signs of neovascular AMD. CFP: colour fundus photography, FAF: fundus autofluorescence, NIR: near infrared reflectance, OCT: optical coherence tomography.

**Figure 4:**
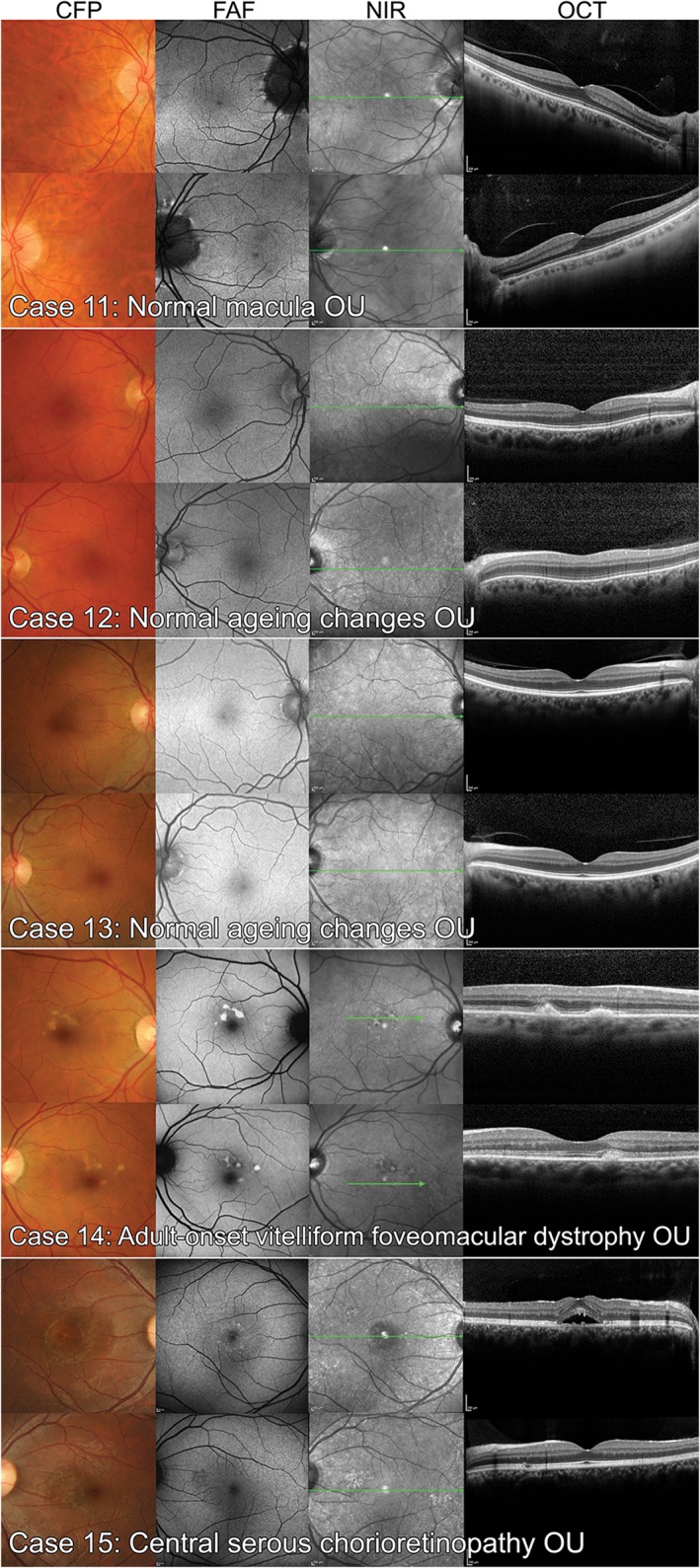
Non‐age‐related macular degeneration case images of the six normal eyes (four with normal ageing changes) and four eyes with other macular diseases used in the study. CFP: colour fundus photography, FAF: fundus autofluorescence, NIR: near infrared reflectance, OCT: optical coherence tomography.

**Figure 5:**
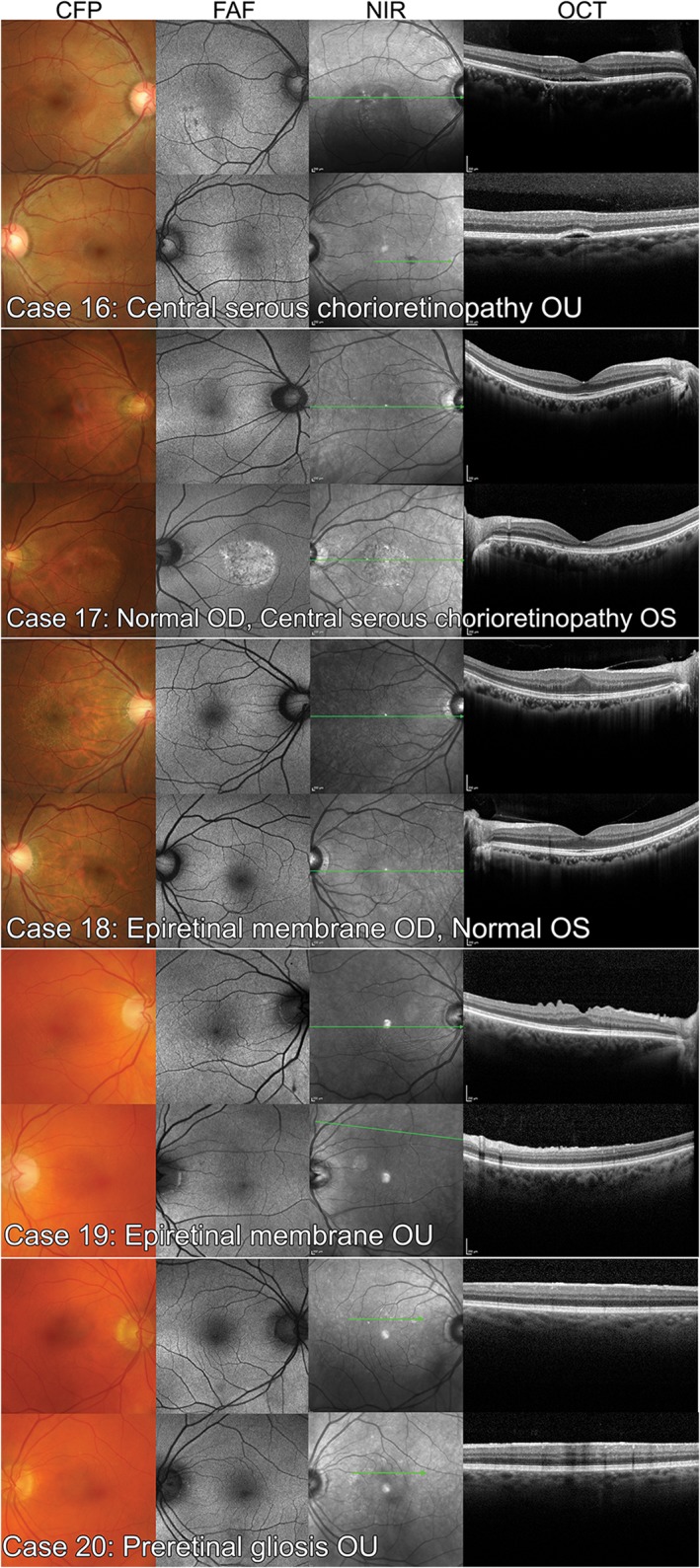
Non‐age‐related macular degeneration case images from the final 10 eyes with other macular diseases: central serous chorioretinopathy and epiretinal membrane. CFP: colour fundus photography, FAF: fundus autofluorescence, NIR: near infrared reflectance, OCT: optical coherence tomography.

Each of the 20 case vignettes was generated using the practice records of real, randomly selected CFEH patients referred for a macular assessment. Cases with incomplete imaging, an equivocal diagnosis or co‐existent ocular disease, were excluded. Images from a total of 40 eyes from 20 patients, 10 with AMD, were included. The AMD eyes (Figures 2 and 3) featured different stages classified as normal ageing to advanced AMD according to the Beckman initiative for macular research classification scheme:^9^ one eye with normal ageing changes, seven with early AMD, 10 with intermediate AMD, one with advanced neovascular AMD, and one with advanced AMD (geographic atrophy). On a case level, this staging corresponded to four cases with early AMD, four cases with intermediate AMD and two cases with advanced AMD. For the non‐AMD eyes, four were normal, four had normal ageing changes, and the remaining 12 had other macular disease (five with epiretinal membrane, five with pachychoroid spectrum disease or central serous chorioretinopathy and two with adult‐onset fovemacular vitelliform dystrophy; Figures 4 and 5).

Colour fundus photographs (CFPs), used in all rounds for each vignette, were acquired using the Kowa WX 3D non‐mydriatic retinal camera (Kowa, http://www.kowa.com; original image size of 2144 × 1424 pixels, 45° field of view). Modified fundus photographs used in rounds two and/or three were composed of red, green and blue digital deconvolutions generated using the ‘split channels’ function of ImageJ, a public domain image processing program (National Institutes of Health, https://imagej.nih.gov/ij/), and adjusted for brightness and contrast. Infrared images acquired with Spectralis Heidelberg Retina Angiograph 2 (HRA2, Heidelberg Engineering, https://www.heidelbergengineering.com/; λ = 815 nm, 30 × 30° field of view, 768 × 768 minimum image size) were provided in tandem with the modified fundus photographs for each case. Uncropped FAF images were also provided during the second and/or final round and acquired using Spectralis HRA2 or Optomap Panoramic 200Tx ultra‐widefield scanning laser ophthalmoscope in ResMax mode (Optos, http://www.optos.com/; 3072 × 3072 pixels, 100° field of view). Participants were free to click on all still images for a magnified view.

For OCT, the macular thickness report for both eyes generated using a Cirrus 5000 HD‐OCT 512 × 128 macular cube (Carl Zeiss Meditec, http://www.zeiss.com/meditec/en_us/home.html) and a video of the Spectralis OCT volume scan for each eye using a 1:1 pixel presentation were provided (scan spacing and density varied based on the judgment of the examining optometrist at the time of assessment, and covered a 20 × 15° to 30 × 25° pattern size, using 19–61 B‐scans, 118–237 μm distance between B‐scans). The video could be replayed any number of times by the participant.

### Statistical analysis

Statistical analyses were performed with SPSS (Version 23; IBM Corporation, Armonk, New York, USA) and figures were generated using GraphPad Prism (Version 6; Graphpad Software, San Diego, California, USA). Demographic data were summarised using descriptive statistics. Responses from the case vignettes were summarised using frequencies of occurrence and contingency tables. Differences between group responses were determined using the chi‐square or Fisher's exact test. Statistically significant differences have been reported at a level of p < 0.05.

The vignettes were primarily used to determine the participants’ diagnoses and management plans. Diagnostic responses for each completed case were evaluated for accuracy and the response provided for each of the three rounds in each case scored one (for AMD) or zero (for ‘normal’ or ‘other macular or retinal disease’). The difference in proportions across the rounds was tested for statistical significance using the Cochran's Q test. Ordinal logistic regression was used to evaluate for an association between high diagnostic accuracy with self‐reported therapeutics endorsement (non‐compulsory licensure to prescribe therapeutic medications: yes or no), and self‐reported experience with AMD. Participants who completed less than eight of the 10 AMD cases (n = 11) were removed from this analysis.

## RESULTS

### Characteristics of the cohort

A total of 1,436 responses were analysed from 81 participants. Each case vignette was completed by 67 to 77 participants in random order (mean of 72). The number of cases completed by each participant was time‐limited and ranged from three to 20 minutes (median time taken to complete each case was four minutes; median of 19 cases per participant). Thirty‐two participants completed all 20 cases (40 per cent completion rate).

Demographic characteristics are summarised in Table 1. Participants were predominantly female (64 per cent) and ranged between 22 and 78 years of age. They were typically engaged in non‐therapeutic, commercial practice (group or solo private practice, or corporate practice) with their highest qualification being a bachelor degree from an Australian university (the minimum required to practice). The majority of participants also indicated that their continuing education history was in excess of requirements set out by the national registration board (68 per cent).^28^ Just under half of all participants (39/81, 48 per cent) indicated that they routinely considered advanced imaging results in the management of AMD.

**Table 1:** Demographic characteristics of the cohort of participants (n = 81)

	Median (IQR)	n	%
A. Full time years of experience	24 (17)		
B. AMD patients per week*	2 (4)		
C. Age*	48 (18.5)		
D. Gender			
Male		29	36%
Female		52	64%
E. Highest level of tertiary education			
Bachelor or equivalent		64	81%
Doctor of Optometry		0	0%
Masters (coursework) or equivalent		8	10%
Masters (research)		3	4%
PhD		4	5%
F. Country of first optometric qualification			
Australia		67	83%
New Zealand		7	9%
Other		7	9%
G. Therapeutics endorsement*			
Yes		24	30%
No		57	70%
H. Continuing education history*			
In excess of registration requirements		53	68%
Not yet meeting the previous year's requirement		25	32%
I. Locum practice*			
Yes		22	28%
No		58	73%
J. Setting of primary practice			
Group private practice		21	26%
Solo private practice		32	40%
Corporate practice		21	26%
Academic institute		5	6%
Other		2	2%

* Denotes findings from a non‐mandatory question. A ‘locum’ describes a temporary position. Therapeutics endorsement describes non‐compulsory licensure to prescribe therapeutic medications.AMD: age‐related macular degeneration, IQR: interquartile range.

Competency in interpreting colour fundus photography was rated as average or above by 97 per cent of participants. Average confidence interpreting other imaging showed greater self‐rated variation: highest for modified retinal photography, followed by OCT and finally FAF. Using ordinal logistic regression, there was no statistically significant association between AMD experience (Table 1) or therapeutics endorsement, with diagnostic accuracy.

### Diagnosis of AMD

**Figure 6:**
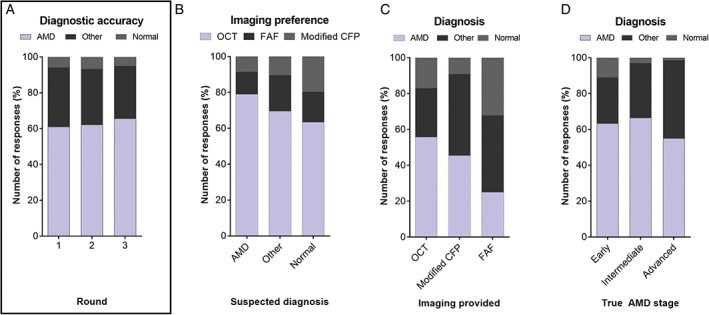
A: Diagnosis (normal, other macular/retinal disease or age‐related macular degeneration [AMD]) as a function of the round when imaging was presented. Round one responses were based on colour fundus photography (CFP) alone. In round two, colour fundus photographs plus the results of one advanced imaging modality were presented. In round three, all imaging results were available for review. The correct diagnosis of all cases was AMD. Diagnostic accuracy improved from 61 per cent in round one to 62 per cent in round two and finally to 66 per cent in round three and these differences were statistically significant (p = 0.004). B: Imaging preference as a function of the suspected diagnosis: participants who suspected AMD more often preferred to see optical coherence tomography (OCT) than those who suspected other macular/retinal disease or a normal macula. In the latter instances, they more often preferred fundus autofluorescence (FAF) and modified retinal photography, respectively. C: Variables affecting the participant's diagnosis included imaging provided and true stage of AMD presented in the worse eye. Using the 127 responses that changed between rounds one and two, OCT was associated with a shift in 43 responses toward the correct diagnosis of AMD, which was higher than modified fundus photography or FAF – 10 and seven instances, respectively. D: There was a greater number diagnosed ‘normal’ in the cases of early AMD and advanced cases were more often misdiagnosed as other macular or retinal diseases across all rounds (averages presented above).

Figure 6A and Table S1 provide a breakdown of the diagnoses chosen after each round of imaging across the 10 AMD case vignettes. Based on the case history, preliminary tests and CFP alone (round one), the 10 cases were accurately described as AMD by 444/728 responses (61 per cent), rather than normal (41/728, six per cent) or other macular/retinal disease (243/728, 33 per cent), yielding a 94 per cent (687/728) total detection level for the presence of any macular pathology. Participants were subsequently queried regarding their advanced imaging preference for each case (Figure 6B). Across all cases, the most popular preference was OCT (546/728, 75 per cent), followed by FAF (112/728, 15 per cent) and modified fundus photography (70/728, 10 per cent). However, OCT was significantly more preferred in cases where AMD was suspected, while FAF was preferential for cases suspected of other macular or retinal diseases and modified retinal photography for normal cases (chi‐square, p < 0.05).

When provided with CFP and one additional imaging modality (round two), 601/728 (83 per cent) responses did not change. Of the 127 changed responses, 60 (47 per cent) corrected the diagnosis to AMD resulting in the diagnostic accuracy of the condition (AMD) by the group as a whole to improve by one per cent only (from 444/728, 61 per cent to 453/728, 62 per cent; Figure 6A). Sixteen (13 per cent) participants changed their diagnosis from other macular or retinal disease to normal or vice versa; 51 (40 per cent) changed away from the correct diagnosis of AMD. Using the 127 responses in which the diagnosis changed between rounds one and two, there was a higher proportion of cases correctly diagnosed with AMD when provided with OCT rather than modified CFP or FAF (Figure 6C).

When all imaging results were provided (round three), diagnostic accuracy for AMD improved by five per cent to 66 per cent (478/728). Of the 151/728 (21 per cent) responses that changed between rounds one and three, 82 (54 per cent) changed toward AMD. Fewer participants (108/728, 15 per cent) changed their diagnosis between rounds two and three and 57/108 (53 per cent) changed toward AMD. The improvement in diagnostic accuracy across each round was statistically significant (Cochran's Q test, p = 0.004).

### False positive diagnoses of AMD

Of the 211 responses for the three case vignettes featuring a normal macula or normal ageing changes, there was a statistically significant change in the false positive rate across each round: seven per cent (15/211) false positive diagnoses of AMD in round one, rising to nine per cent (18/211) in round two, and 12 per cent (26/211) in round three (Cochran's Q test, p = 0.044). For case vignettes 14–20 featuring other macular disease, 18 per cent (90/497) of responses erroneously indicated a diagnosis of AMD in round one, 20 per cent (101/497) in round two and 21 per cent (104/497) in round three. The difference between rounds was not statistically significant (Cochran's Q test, p = 177). The distribution of misdiagnoses across each of the three rounds varied significantly with the case presented (Table S2; chi‐square, p < 0.001). Interestingly, case 17 (an instance of unilateral central serous chorioretinopathy) was most commonly misdiagnosed as AMD by 48 per cent (35/73) of respondents in round one (based on the case history and colour fundus photography alone).

### Case effects on the diagnosis of AMD

When the true stage of AMD (using the worse eye) was considered, there was also a statistically significant difference between the spread of misdiagnoses (chi‐square, p < 0.001; Figure 6D); in the cases of early AMD, a significantly greater number of ‘normal’ responses occurred. Cases of intermediate AMD were most often accurately identified as AMD, while cases of advanced AMD were more often misdiagnosed as other macular or retinal disease. This effect was preserved across the spread of responses for all three rounds (averages presented in Figure 6D).

**Figure 7:**
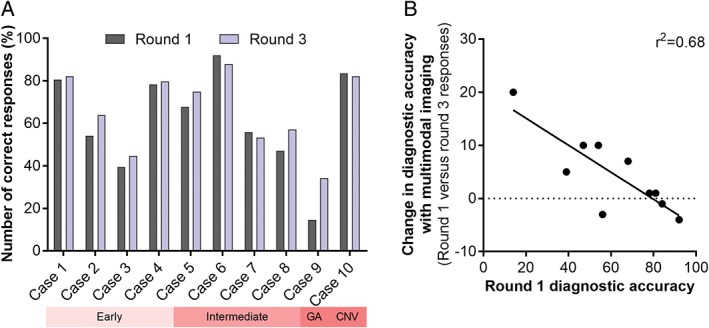
A: Diagnostic accuracy as a function of the case type and round. Round one responses were based on colour fundus photography alone, while round three followed the review of colour fundus photographs and all of the advanced imaging findings. Across the cohort, 7/10 cases showed an improvement in diagnostic accuracy ranging from one per cent to 20 per cent. Three out of 10 cases (cases 6, 7 and 10) showed a decrease in diagnostic accuracy between minus one per cent and minus four per cent. The decrements were associated with cases that had high diagnostic accuracy using colour fundus photography alone. B: Scatterplot demonstrating the correlation between round one diagnostic accuracy using colour fundus photography and the improvement with multimodal imaging. CNV: choroidal neovascularisation, GA: geographical atrophy.

Following evaluation of broad diagnostic accuracy using the total dataset, we wanted to assess responses on a case level. Diagnostic accuracy of AMD ranged between 11/76 (14 per cent) to 69/75 (92 per cent) for each case. Using one additional imaging modality, 2/10 cases showed a net improvement in diagnostic accuracy compared to the diagnosis based on CFP alone. A majority (7/10) of cases showed an improvement when all imaging results were provided (Figure 7A). Between rounds two and three the change in diagnostic accuracy ranged between −1 to 10 per cent; 6/10 cases showed an improvement. There was a statistically significant correlation between the average diagnostic accuracy of the case using CFP (round one responses) and the improvement with multimodal imaging (Figure 7B, r^2^ = 0.68, p < 0.005).

### Signs and staging of AMD

For any instance in which the case was diagnosed correctly with AMD (n = 444), the participant was subsequently asked to identify the AMD signs and stage present. In most cases, the suspected AMD stage of the participants was consistent with the correct stage or represented an underestimate of disease severity, for example intermediate AMD mis‐staged as early (Table S1).

For pigmentary changes, signs of neovascular AMD and geographic atrophy, there was an overall significant association between the signs of AMD identified and the AMD stage across all rounds using the responses from either eye in a direction consistent with the Beckman initiative for macular research clinical classification scheme (BIMR; Table 2).^9^

**Table 2:** Correlation matrix relating age‐related macular degeneration (AMD) signs and staging from 888 round one responses; answers inconsistent with the evidence‐base appear bold

	Normal ageing*	Early*	Intermediate*	Advanced*
Drusen	42	496	256	14
Reticular pseudodrusen	13	25	31	0
Pigmentary changes	**11**	**126**	111	8
Geographic atrophy	**2**	**14**	**20**	2
Signs of neovascular AMD	**0**	**17**	**12**	8

* Stage of AMD according to the Beckman classification definitions, in which persons with small drusen only should be considered to have normal ageing changes. Persons with medium drusen only have early AMD while large drusen and/or pigmentary changes associated with at least medium drusen signify intermediate AMD. Advanced AMD includes lesions due to geographic atrophy or choroidal neovascularisation.

However, there was also considerable disparity between the signs identified and the AMD stage with the BIMR.^9^ This recent AMD clinical classification scheme stipulates that pigmentary changes associated with at least medium drusen should be classified as intermediate AMD. Contrary to that, in round one, there were 11 (one per cent) and 126 (14 per cent) instances (out of a total of 888 responses using data from both eyes) in which pigmentary changes were identified, although graded with normal ageing changes or early AMD, respectively. Similarly and contrary to the evidence, geographic atrophy was identified in two, 14 and 20 eyes (up to two per cent) classified as normal ageing changes, early AMD and intermediate AMD. Signs of neovascular AMD were flagged in 17 (two per cent) and 12 eyes (one per cent) classified with early and intermediate AMD, respectively.

There was no statistically significant association between the signs of AMD identified (drusen, pigmentary changes, reticular pseudodrusen, geographic atrophy or signs of neovascular AMD) in either eye based on the type of imaging provided in round two (chi‐square, p > 0.05).

### Management of AMD

The management plan is provided in Table S3 and was classified dichotomously as the intention of participants to review or refer to another health professional. Based on all round one responses, there was a slight majority preference overall to review (418/728, 57 per cent) rather than refer (310/728, 43 per cent). This bias toward optometric reviewing decreased (the preference to refer increased) with both single (average 390/728, 54 per cent) and multimodal imaging (383/728, 53 per cent).

**Figure 8:**
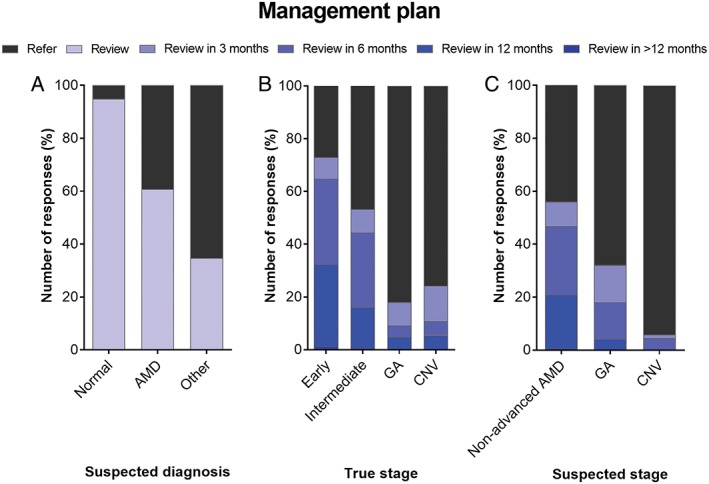
Factors affecting the participant's management plan using averaged response proportions across the three rounds. A: Suspected diagnosis: as a group, participants indicated that they would most often review the case if they identified it as normal, followed by age‐related macular degeneration (AMD) then other macular or retinal disease. B: True stage of the AMD case presented, using the worse eye: review tendency was associated with lower rated disease severity. C: Participant's suspected AMD stage: a majority 94 per cent of participants would refer if signs of neovascular AMD were present in either eye. CNV: choroidal neovascularisation, GA: geographic atrophy.

There was a statistically significant difference in the management plan with the suspected diagnosis, across all imaging rounds (chi‐square, p < 0.001; Figure 8A). Not surprisingly, the participants more often indicated they would review the case if they diagnosed it as normal, followed by AMD then other macular or retinal disease. This response pattern was maintained across all rounds. Participants who diagnosed cases correctly as AMD indicated that they would review it in 279/458 (61 per cent) of instances on average across the three rounds.

Similarly, the management plan differed significantly with the true stage of AMD presented in the case (using the worse eye; Figure 8B). Early AMD cases were more likely to be reviewed, followed by intermediate AMD then advanced AMD cases (chi‐square, p < 0.001). This effect was maintained across all rounds and also consistent (and more distinct) with the participant's suspected stage of AMD right eye and left eye (Figure 8C). One hundred per cent of all responses suspected of advanced AMD were referred. The presence of AMD signs was also significantly linked to the management plan: signs of neovascular AMD (chi‐square, p < 0.001), geographic atrophy (p < 0.05) and pigmentary changes (p < 0.001) were associated with referral and a majority 94 per cent of participants would refer if signs of neovascular AMD were present in either eye.

If participants indicated that they would review the case, most stipulated a review period of six months (183/397, 46 per cent average across all rounds), followed by 12 months (143/397, 36 per cent), three months (66/397, 17 per cent) and rarely >12 months (5/397, one per cent). For all instances where referral was selected, referral to a private ophthalmologist (304/331, 92 per cent) rather than another optometrist (8/331, two per cent), or public ophthalmologist (19/331, six per cent) was the most frequent response. This order of preferences was independent of the round in which the responses were provided during the case vignette.

## DISCUSSION

There has recently been a clinical shift toward the integration of advanced imaging technologies, especially OCT, into routine optometric assessment and management.^29^, ^30^ Our work highlights several trends regarding the preferences and utility of imaging in AMD among a subset of practising optometrists in Australia (primarily New South Wales). Our data reveals that the cohort prefers OCT over other modalities, although the strength of this preference apparently varies with the suspected diagnosis (normal, AMD or other macular or retinal disease).

The influence of suspected diagnosis on imaging preference reflects the core principles of advanced imaging: OCT represents the most ‘indispensable’ of the techniques to clinical practice, evidenced by the greatest improvement in AMD diagnostic accuracy compared to other modalities. By comparison, FAF is a more targeted modality specific to retinal pigment epithelium health relevant to AMD and other disorders, although may be of limited use in the assessment of a ‘normal’ macula.

In our cohort of 81 practising optometrists, the diagnostic accuracy of AMD cases using CFP was 61 per cent. Approximately one‐fifth of presentations with other macular diseases may also be misdiagnosed as AMD. Reassuringly, the AMD cases were seldom misdiagnosed as normal (six per cent in round one) indicating a comparatively high detection rate for the presence or absence of any macular pathology. Diagnostic accuracy of AMD improved by a modest five per cent using advanced imaging but was also associated with an increase in false positives and increased tendency to refer, identifying a knowledge gap regarding the interpretation of ocular imaging among the cohort of practising optometrists.

Most interestingly, our findings suggested that the improvement in diagnostic accuracy with imaging may also be maximal with OCT and directly related to the diagnostic accuracy achieved using CFP alone, that is the greatest improvement in diagnostic accuracy (up to 20 per cent) was observed in cases that scored the poorest in round one. Accordingly, eye‐care professionals in practice should be conscious of these biases and pursue further imaging judiciously. Cases where the diagnosis is equivocal or inconclusive based on CFP alone may be more suitable for advanced imaging. The decision to apply advanced imaging technologies should also take into consideration its impact on clinical decision making and patient outcomes.^31^

Our data also support the notion that optometrists in practice follow a signs‐and‐stage‐based approach to the diagnosis of AMD. Practising optometrists in our cohort were more capable of accurately identifying early and intermediate AMD rather than advanced presentations, which reflects the cases optometrists are likely to encounter. In general, there was also a tendency for participants to under‐stage the case presentation. When the case was accurately identified as AMD, most participants displayed an evidence‐based approach to staging based on the signs identified. However, up to 14 per cent of practising optometrists may not be aware or are not accurately applying the recent clinical classification scheme^9^ to AMD cases (pigmentary changes misidentified as early AMD).

Interestingly, there was also no statistically significant difference in the nature of AMD signs identified with the type of imaging provided. This finding runs contrary to the evidence base, which suggests that certain modalities are better suited to the detection of certain AMD phenotypes, for example FAF for geographic atrophy,^32^ blue or infrared reflectance imaging for reticular pseudodrusen.^33^ One possibility is that the case vignettes of our study may be insensitive to measuring the hypothesised change. More likely, this data may be reminiscent of the relatively rapid dissemination of imaging technologies into clinical practice and a lack of formal, disease‐specific training on their utility. Training, education programs and professional competencies need to accurately and effectively reflect the improvement opportunities relating to the application of advanced imaging in clinical practice.

Evidently, management practices (plans to review or refer each case) especially in intermediate AMD vary considerably between optometrists, although they trend toward 6–12 monthly review and referral to an ophthalmologist. Of interest, our data also showed 12/73 instances (16 per cent) in which case 10 of neovascular AMD would be reviewed (rather than referred) due to an error in staging or diagnosis, despite the results of advanced imaging. This would preclude treatment and has significant implications on patient care. Thus, further investigation into the diagnosis and management of advanced AMD by optometrists is recommended. Practice variation is not unique to optometry and although practice variation may not always be inappropriate, it has been used as a surrogate metric for practice quality.^26^ Inappropriate practice variation such as regarding management can lead to undesirable outcomes, including harm to the patient and unnecessary service burden, for which tele‐ophthalmology, collaborative and innovative care models may be useful.^21^, ^23^, ^34^ On the other hand, all cases suspected of advanced AMD were referred.

### Limitations

Although action in clinical practice is difficult to measure,^35^ case vignettes represent the most valid, robust and relevant strategy for measuring clinical competency and evaluating point‐of‐care clinical decisions.^36^, ^37^, ^38^ They are also practical, applicable to our research question, relatively inexpensive (compared to standardised patients or chart abstraction) and control for patient variation (unlike claims or insurance history or outcome metrics).^26^, ^37^, ^38^, ^39^, ^40^

Recommendations relating to the successful application of case vignettes have been detailed elsewhere but, briefly, should feature a realistic level of clinical complexity and timing in order to maximise their validity.^26^, ^36^, ^38^ Our case vignettes were created from a random selection of real clinical cases in order to reflect a realistic level of complexity that practising clinicians are likely to encounter. However, patients encountered in a general optometric setting are also typically healthy, limiting the generalisability of our findings.

We were also interested in the individual effect of different imaging technologies on diagnostic accuracy in clinical practice, and thus prospectively randomised the imaging presented in round two. Each case vignette was also pilot‐tested carefully by CFEH optometrists and participants were specifically advised to answer as they would ordinarily do in everyday practice. However, this case mix was consequently dominated by early and intermediate AMD and included only one case each of geographic atrophy and choroidal neovascularisation. Thus, it may not reflect the presentations where advanced imaging is most likely to be valued or clinically indicated. The same methodology precluded the calculation of true sensitivity or specificity values, such as is available elsewhere.^22^, ^41^, ^42^, ^43^, ^44^, ^45^, ^46^, ^47^, ^48^, ^49^, ^50^, ^51^, ^52^, ^53^

Other possible confounding factors in our study that might affect the quality and generalisability of our data include: (i) sampling bias and the tendency to attract participants with a specialty interest in ocular disease or AMD research; (ii) variations in the comfort of participants (especially those who were older) with using computers; (iii) cueing – by asking first for the signs of AMD observed prior to the stage, leading to an overestimate of competency; (iv) the Hawthorne effect – social desirability bias relating to participants knowing that their clinical performance was being evaluated; and (v) satisficing – the phenomenon by which participants may perform the minimum possible in order to achieve a goal, which typically occurs if the task is too difficult or if participant motivation is low.

Data derived from case vignettes, such as ours, cannot be assumed to translate wholly to habitual practice actions, which may be influenced by a range of other variables (such as communication, clinical examination skills and instrument‐specific training). For instance, the detection and management of neovascular AMD may be supported by the case history (new symptoms of distortions) and stereoscopic examination of the macula. Although we queried participants regarding their routine consideration of advanced imaging results in the management of AMD, this appears not to translate to familiarity in interpretation. Furthermore, our results do not provide insight into optometric management practices regarding nutritional supplements for AMD or the clinical reasoning behind the participants’ decisions.

Specifically regarding management congruency, case vignettes fail to take into account such external factors as patient values, cost and insurance considerations, practice level influences or time constraints which may influence the actual management plan. Other authors have emphasised the known inaccuracies of case vignettes in reporting treatment plans,^54^ which limit the generalisability of our findings.

### Future directions

As recommended by Shah et al.,^54^ the purpose of our study was to be descriptive rather than critical. Future studies are suggested in order to further highlight the commitment of eye‐care professions to promote a high standard. Measurements of clinical practice, such as ours, may be used: (i) to evaluate and describe the current standard of eye‐care, which may have clinico‐legal implications; (ii) to encourage confidence in the eye‐care professions; (iii) to determine future priorities in undergraduate or continuing postgraduate education and to set a realistic groundwork for evidence‐based practice and minimum standards of competency; and (iv) to enable future measurements of the impact of change strategies.^54^

It was beyond the scope of this study to explore differences between groups of eye‐care professionals based on their training or even country of practice, or to elucidate the specific reasons behind limitations in practice. As with others,^26^, ^36^ we also recommend further study of practice variation and ongoing efforts in order to better understand contributing factors and their effects on patient outcomes.

## CONCLUSION

Our data highlight that the effect of advanced imaging on diagnostic accuracy in AMD by a cohort of practising clinicians is small at five per cent and is associated with a higher intention to refer. It also suggests an apparent lack of training among participants regarding the specific interpretation of imaging in AMD and provides unique insight into factors which may be useful for improving the current standard of care.

## Supporting information


**Figure S1**. Screenshot illustrating the instructions provided to each participant at the beginning of the survey.

**Table S1**. Distribution of diagnostic and staging responses across all 10 AMD cases.

**Table S2**. Distribution of diagnostic responses across the 10 non‐AMD cases.

**Table S3**. Management responses across all 10 AMD case vignettes. The designated case stage (first column) indicates the case severity in the worse eye.
