# Supplementary material for: Advanced imaging for the diagnosis of age‐related macular degeneration: a case vignettes study
Source: Clin Exp Optom. 2017 Oct 9;101(2):243–54. doi: 10.1111/cxo.12607 (PMC5873408; doi:10.1111/cxo.12607)

**Figure S1.** Screenshot illustrating the instructions provided to each participant at the beginning of the survey.


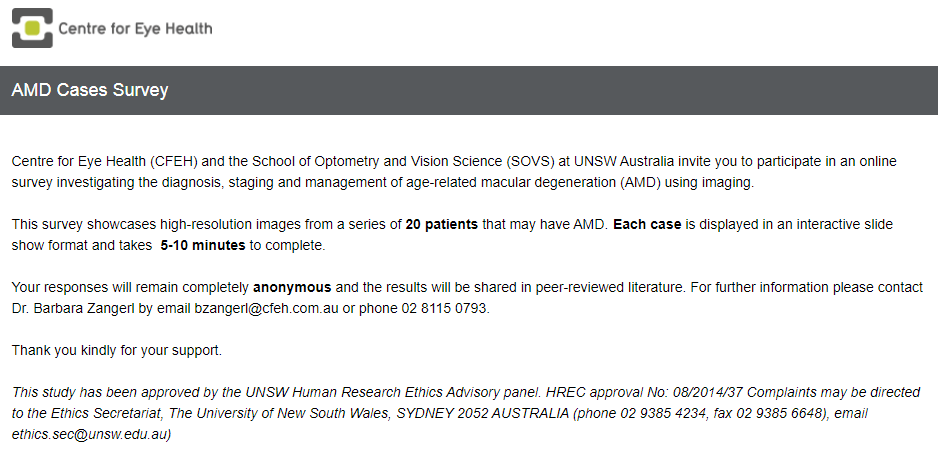

Supplement: Supplementary file 1 — Figure S1. Screenshot illustrating the instructions provided to each participant at the beginning of the survey. [file CXO-101-243-s004.docx]
