# Supplementary material for: Advanced imaging for the diagnosis of age‐related macular degeneration: a case vignettes study
Source: Clin Exp Optom. 2017 Oct 9;101(2):243–54. doi: 10.1111/cxo.12607 (PMC5873408; doi:10.1111/cxo.12607)
Supplement: Supplementary file 2 — Table S1. Distribution of diagnostic and staging responses across all 10 AMD cases. [file CXO-101-243-s002.docx]

**Table S1. Distribution of diagnostic and staging responses across all ten AMD cases**

|  |  |  |  |  |  |  |  | Right eye (OD) | | | | | | | | Left eye (OS) | | | | | | | |
| --- | --- | --- | --- | --- | --- | --- | --- | --- | --- | --- | --- | --- | --- | --- | --- | --- | --- | --- | --- | --- | --- | --- | --- |
|  |  | Normal | | Other † | | AMD | | Normal ageing | | Early AMD | | Intermediate AMD | | Advanced AMD | | Normal ageing | | Early AMD | | Intermediate AMD | | Advanced AMD | |
| Case 1 | CFP only | 9 | (13%) | 4 | (6%) | ***54*** | ***(81%)*** | 2 | (3%) | ***46*** | ***(69%)*** | 6 | (9%) | 0 | (0%) | 2 | (3%) | ***45*** | ***(67%)*** | 7 | (10%) | 0 | (0%) |
|  | CFP+1 imaging | 7 | (10%) | 7 | (10%) | ***53*** | ***(79%)*** | 2 | (3%) | ***47*** | ***(70%)*** | 4 | (6%) | 0 | (0%) | 2 | (3%) | ***46*** | ***(69%)*** | 5 | (7%) | 0 | (0%) |
|  | CFP+all imaging | 2 | (3%) | 10 | (15%) | ***55*** | ***(82%)*** | 4 | (6%) | ***45*** | ***(67%)*** | 6 | (9%) | 0 | (0%) | 3 | (4%) | ***44*** | ***(66%)*** | 8 | (12%) | 0 | (0%) |
| Case 2 | CFP only | 10 | (14%) | 23 | (32%) | ***39*** | ***(54%)*** | 4 | (6%) | ***32*** | ***(44%)*** | 3 | (4%) | 0 | (0%) | 4 | (6%) | ***31*** | ***(43%)*** | 3 | (4%) | 1 | (1%) |
|  | CFP+1 imaging | 11 | (15%) | 22 | (31%) | ***39*** | ***(54%)*** | 2 | (3%) | ***34*** | ***(47%)*** | 3 | (4%) | 0 | (0%) | 3 | (4%) | ***33*** | ***(46%)*** | 3 | (4%) | 0 | (0%) |
|  | CFP+all imaging | 15 | (21%) | 11 | (15%) | ***46*** | ***(64%)*** | 5 | (7%) | ***38*** | ***(53%)*** | 3 | (4%) | 0 | (0%) | 4 | (6%) | ***39*** | ***(54%)*** | 3 | (4%) | 0 | (0%) |
| Case 3 | CFP only | 1 | (1%) | *45* | *(59%)* | **30** | **(39%)** | ***17*** | ***(22%)*** | 13 | (17%) | 0 | (0%) | 0 | (0%) | 0 | (0%) | ***22*** | ***(29%)*** | 8 | (11%) | 0 | (0%) |
|  | CFP+1 imaging | 3 | (4%) | *45* | *(59%)* | **28** | **(37%)** | ***16*** | ***(21%)*** | 11 | (14%) | 1 | (1%) | 0 | (0%) | 1 | (1%) | ***22*** | ***(29%)*** | 4 | (5%) | 1 | (1%) |
|  | CFP+all imaging | 3 | (4%) | *39* | *(51%)* | **34** | **(45%)** | ***20*** | ***(26%)*** | 14 | (18%) | 0 | (0%) | 0 | (0%) | 0 | (0%) | ***28*** | ***(37%)*** | 6 | (8%) | 0 | (0%) |
| Case 4 | CFP only | 10 | (14%) | 6 | (8%) | ***58*** | ***(78%)*** | 8 | (11%) | ***47*** | ***(64%)*** | 3 | (4%) | 0 | (0%) | 7 | (9%) | ***49*** | ***(66%)*** | 2 | (3%) | 0 | (0%) |
|  | CFP+1 imaging | 13 | (18%) | 6 | (8%) | ***55*** | ***(74%)*** | 8 | (11%) | ***44*** | ***(59%)*** | 3 | (4%) | 0 | (0%) | 7 | (9%) | ***47*** | ***(64%)*** | 1 | (1%) | 0 | (0%) |
|  | CFP+all imaging | 9 | (12%) | 6 | (8%) | ***59*** | ***(80%)*** | 6 | (8%) | ***51*** | ***(69%)*** | 2 | (3%) | 0 | (0%) | 6 | (8%) | ***52*** | ***(70%)*** | 1 | (1%) | 0 | (0%) |
| Case 5 | CFP only | 3 | (4%) | 19 | (28%) | ***46*** | ***(68%)*** | 3 | (4%) | 20 | (29%) | ***22*** | ***(32%)*** | 1 | (1%) | 3 | (4%) | *22* | *(32%)* | **20** | **(29%)** | 1 | (1%) |
|  | CFP+1 imaging | 0 | (0%) | 24 | (35%) | ***44*** | ***(65%)*** | 2 | (3%) | 18 | (26%) | ***23*** | ***(34%)*** | 1 | (1%) | 2 | (3%) | 20 | (29%) | ***21*** | ***(31%)*** | 1 | (1%) |
|  | CFP+all imaging | 2 | (3%) | 15 | (22%) | ***51*** | ***(75%)*** | 2 | (3%) | 17 | (25%) | ***32*** | ***(47%)*** | 0 | (0%) | 2 | (3%) | 20 | (29%) | ***29*** | ***(43%)*** | 0 | (0%) |
| Case 6 | CFP only | 1 | (1%) | 5 | (7%) | ***69*** | ***(92%)*** | 1 | (1%) | *34* | *(45%)* | **32** | **(43%)** | 2 | (3%) | 1 | (1%) | *33* | *(44%)* | ***33*** | **(44%)** | 2 | (3%) |
|  | CFP+1 imaging | 3 | (4%) | 4 | (5%) | ***68*** | ***(91%)*** | 2 | (3%) | *32* | *(43%)* | ***32*** | ***(43%)*** | 2 | (3%) | 2 | (3%) | 30 | (40%) | ***34*** | **(45%)** | 2 | (3%) |
|  | CFP+all imaging | 1 | (1%) | 8 | (11%) | ***66*** | ***(88%)*** | 1 | (1%) | 26 | (35%) | ***38*** | ***(51%)*** | 1 | (1%) | 1 | (1%) | 26 | (35%) | ***37*** | **(49%)** | 2 | (3%) |
| Case 7 | CFP only | 3 | (4%) | *31* | (40%) | ***43*** | ***(56%)*** | 5 | (6%) | 17 | (22%) | ***21*** | ***(27%)*** | 0 | (0%) | 5 | (6%) | 17 | (22%) | ***21*** | ***(27%)*** | 0 | (0%) |
|  | CFP+1 imaging | 5 | (6%) | *30* | (39%) | ***42*** | ***(55%)*** | 3 | (4%) | *20* | *(26%)* | **19** | **(25%)** | 0 | (0%) | 3 | (4%) | 19 | (25%) | ***20*** | ***(26%)*** | 0 | (0%) |
|  | CFP+all imaging | 1 | (1%) | *35* | (45%) | ***41*** | ***(53%)*** | 1 | (1%) | 18 | (23%) | ***21*** | ***(27%)*** | 1 | (1%) | 1 | (1%) | 18 | (23%) | ***21*** | ***(27%)*** | 1 | (1%) |
| Case 8 | CFP only | 1 | (1%) | *36* | *(51%)* | **33** | **(47%)** | 1 | (1%) | *17* | *(24%)* | **15** | **(21%)** | 0 | (0%) | 1 | (1%) | 14 | (20%) | ***17*** | ***(24%)*** | 1 | (1%) |
|  | CFP+1 imaging | 3 | (4%) | *31* | (44%) | ***36*** | ***(51%)*** | 3 | (4%) | *18* | *(26%)* | **15** | **(21%)** | 0 | (0%) | 2 | (3%) | 14 | (20%) | ***19*** | ***(27%)*** | 1 | (1%) |
|  | CFP+all imaging | 1 | (1%) | *29* | (41%) | ***40*** | ***(57%)*** | 2 | (3%) | *21* | *(30%)* | **17** | **(24%)** | 0 | (0%) | 2 | (3%) | 16 | (23%) | ***21*** | ***(30%)*** | 1 | (1%) |
| Case 9 | CFP only | 2 | (3%) | *63* | *(83%)* | **11** | **(14%)** | 1 | (1%) | *9* | *(12%)* | **1** | **(1%)** | 0 | (0%) | 0 | (0%) | *10* | *(13%)* | 1 | (1%) | **0** | **(0%)** |
|  | CFP+1 imaging | 1 | (1%) | *48* | *(63%)* | **27** | **(36%)** | 2 | (3%) | *16* | *(21%)* | **7** | **(9%)** | 2 | (3%) | 2 | (3%) | 8 | (11%) | *11* | *(14%)* | **6** | **(8%)** |
|  | CFP+all imaging | 0 | (0%) | *50* | *(66%)* | **26** | **(34%)** | 4 | (5%) | *14* | *(18%)* | **7** | **(9%)** | 1 | (1%) | 1 | (1%) | 6 | (8%) | *14* | *(18%)* | **5** | **(7%)** |
| Case 10 | CFP only | 1 | (1%) | 11 | (15%) | ***61*** | ***(84%)*** | 7 | (10%) | *25* | *(34%)* | 24 | (33%) | **5** | **(7%)** | 0 | (0%) | *31* | *(42%)* | **28** | **(38%)** | 2 | (3%) |
|  | CFP+1 imaging | 1 | (1%) | 11 | (15%) | ***61*** | ***(84%)*** | 3 | (4%) | *25* | *(34%)* | 24 | (33%) | **9** | **(12%)** | 1 | (1%) | *30* | *(41%)* | ***30*** | ***(41%)*** | 0 | (0%) |
|  | CFP+all imaging | 0 | (0%) | 13 | (18%) | ***60*** | ***(82%)*** | 1 | (1%) | 15 | (21%) | *26* | *(36%)* | **18** | **(25%)** | 1 | (1%) | 24 | (33%) | ***35*** | ***(48%)*** | 0 | (0%) |

† truncated from other macular or retinal disease; CFP, colour fundus photography. Evidence-based answers appear bold, while the most popular response appears in italics.
