# Supplementary material for: Advanced imaging for the diagnosis of age‐related macular degeneration: a case vignettes study
Source: Clin Exp Optom. 2017 Oct 9;101(2):243–54. doi: 10.1111/cxo.12607 (PMC5873408; doi:10.1111/cxo.12607)
Supplement: Supplementary file 3 — Table S2. Distribution of diagnostic responses across the 10 non‐AMD cases. [file CXO-101-243-s003.docx]

**Table S2. Distribution of diagnostic responses across the ten non-AMD cases**

|  |  |  |  |  |  |  |  |
| --- | --- | --- | --- | --- | --- | --- | --- |
|  |  | Normal | | Other † | | AMD | |
| Case 11 | CFP only | ***51*** | ***(72%)*** | 20 | (28%) | 0 | (0%) |
| Normal macula OU | CFP+1 imaging | ***35*** | ***(49%)*** | *35* | *(49%)* | 1 | (1%) |
|  | CFP+all imaging | **29** | **(41%)** | *41* | *(58%)* | 1 | (1%) |
| Case 12 | CFP only | ***57*** | ***(83%)*** | 8 | (12%) | 4 | (6%) |
| Normal ageing changes OU | CFP+1 imaging | ***45*** | ***(65%)*** | 16 | (23%) | 8 | (12%) |
|  | CFP+all imaging | ***36*** | ***(52%)*** | 18 | (26%) | 15 | (22%) |
| Case 13 | CFP only | ***37*** | ***(52%)*** | 23 | (32%) | 11 | (15%) |
| Normal ageing changes OU | CFP+1 imaging | ***40*** | ***(56%)*** | 22 | (31%) | 9 | (13%) |
|  | CFP+all imaging | ***32*** | ***(45%)*** | 29 | (41%) | 10 | (14%) |
| Case 14 | CFP only | 2 | (3%) | ***50*** | ***(72%)*** | 17 | (25%) |
| Adult-onset vitelliform foveomacular dystrophy OU | CFP+1 imaging | 1 | (1%) | **33** | **(48%)** | *35* | *(51%)* |
|  | CFP+all imaging | 2 | (3%) | **30** | **(43%)** | *37* | *(54%)* |
| Case 15 | CFP only | 1 | (1%) | ***64*** | ***(94%)*** | 3 | (4%) |
| Central serous chorioretinopathy OU | CFP+1 imaging | 0 | (0%) | ***65*** | ***(96%)*** | 3 | (4%) |
|  | CFP+all imaging | 0 | (0%) | ***65*** | ***(96%)*** | 3 | (4%) |
| Case 16 | CFP only | 10 | (14%) | ***55*** | ***(79%)*** | 5 | (7%) |
| Central serous chorioretinopathy OU | CFP+1 imaging | 2 | (3%) | ***64*** | ***(91%)*** | 4 | (6%) |
|  | CFP+all imaging | 3 | (4%) | ***64*** | ***(91%)*** | 3 | (4%) |
| Case 17 | CFP only | 0 | (0%) | ***38*** | ***(52%)*** | 35 | (48%) |
| Normal OD, Central serous chorioretinopathy OS | CFP+1 imaging | 0 | (0%) | ***41*** | ***(56%)*** | 32 | (44%) |
|  | CFP+all imaging | 0 | (0%) | ***37*** | ***(51%)*** | 36 | (49%) |
| Case 18 | CFP only | 0 | (0%) | ***59*** | ***(80%)*** | 15 | (20%) |
| Epiretinal membrane OD, Normal OS | CFP+1 imaging | 0 | (0%) | ***63*** | ***(85%)*** | 11 | (15%) |
|  | CFP+all imaging | 2 | (3%) | ***60*** | ***(81%)*** | 12 | (16%) |
| Case 19 | CFP only | 24 | (32%) | ***41*** | ***(54%)*** | 11 | (14%) |
| Epiretinal membrane OU | CFP+1 imaging | 2 | (3%) | ***64*** | ***(84%)*** | 10 | (13%) |
|  | CFP+all imaging | 1 | (1%) | ***70*** | ***(92%)*** | 5 | (7%) |
| Case 20 | CFP only | *56* | *(84%)* | **7** | **(10%)** | 4 | (6%) |
| Preretinal gliosis OU | CFP+1 imaging | *48* | *(72%)* | **13** | **(19%)** | 6 | (9%) |
|  | CFP+all imaging | *38* | *(57%)* | **21** | **(31%)** | 8 | (12%) |

† truncated from other macular or retinal disease; CFP, colour fundus photography. Evidence-based answers appear bold, while the most popular response appears in italics.
