# Supplementary material for: Advanced imaging for the diagnosis of age‐related macular degeneration: a case vignettes study
Source: Clin Exp Optom. 2017 Oct 9;101(2):243–54. doi: 10.1111/cxo.12607 (PMC5873408; doi:10.1111/cxo.12607)
Supplement: Supplementary file 4 — Table S3. Management responses across all 10 AMD case vignettes. The designated case stage (first column) indicates the case severity in the worse eye. [file CXO-101-243-s001.docx]

**Table S3. Management responses across all ten AMD case vignettes.** The designated case stage (first column) indicates the case severity in the worse eye.

|  |  | Review period | | | | | | | | | | Referral plan | | | | | | | |
| --- | --- | --- | --- | --- | --- | --- | --- | --- | --- | --- | --- | --- | --- | --- | --- | --- | --- | --- | --- |
|  |  | 3 months | | 6 months | | 12 months | | >12 months | | Total who would review | | Total who would refer | | Another optometrist | | Private ophthalmologist | | Public ophthalmologist | |
| Case 1 | CFP only | 5 | (7%) | 24 | (36%) | *27* | *(40%)* | 1 | (1%) | *57* | *(85%)* | 10 | (15%) | 2 | (3%) | *5* | *(7%)* | 3 | (4%) |
| Early | CFP+1 imaging | 4 | (6%) | 17 | (25%) | *27* | *(40%)* | 0 | (0%) | *48* | *(72%)* | 19 | (28%) | 2 | (3%) | *12* | *(18%)* | 5 | (7%) |
|  | CFP+all imaging | 4 | (6%) | 16 | (24%) | *26* | *(39%)* | 0 | (0%) | *46* | *(69%)* | 21 | (31%) | 1 | (1%) | *17* | *(25%)* | 3 | (4%) |
| Case 2 | CFP only | 6 | (8%) | *30* | *(42%)* | 14 | (19%) | 0 | (0%) | *50* | *(69%)* | 22 | (31%) | 3 | (4%) | *17* | *(24%)* | 2 | (3%) |
| Early | CFP+1 imaging | 7 | (10%) | *28* | *(39%)* | 21 | (29%) | 1 | (1%) | *57* | *(79%)* | 15 | (21%) | 2 | (3%) | *13* | *(18%)* | 0 | (0%) |
|  | CFP+all imaging | 5 | (7%) | *31* | *(43%)* | 22 | (31%) | 1 | (1%) | *59* | *(82%)* | 13 | (18%) | 1 | (1%) | *12* | *(17%)* | 0 | (0%) |
| Case 3 | CFP only | 9 | (12%) | *16* | *(21%)* | 7 | (9%) | 0 | (0%) | 32 | (42%) | *44* | *(58%)* | 0 | (0%) | *42* | *(55%)* | 2 | (3%) |
| Early | CFP+1 imaging | 12 | (16%) | *16* | *(21%)* | 9 | (12%) | 0 | (0%) | 37 | (49%) | *39* | *(51%)* | 1 | (1%) | *35* | *(46%)* | 3 | (4%) |
|  | CFP+all imaging | 9 | (12%) | *21* | *(28%)* | 11 | (14%) | 0 | (0%) | *41* | *(54%)* | 35 | (46%) | 0 | (0%) | *33* | *(43%)* | 2 | (3%) |
| Case 4 | CFP only | 4 | (5%) | 28 | (38%) | *34* | *(46%)* | 2 | (3%) | *68* | *(92%)* | 6 | (8%) | 2 | (3%) | 1 | (1%) | *3* | *(4%)* |
| Early | CFP+1 imaging | 2 | (3%) | 27 | (36%) | *37* | *(50%)* | 3 | (4%) | *69* | *(93%)* | 5 | (7%) | 0 | (0%) | *3* | *(4%)* | 2 | (3%) |
|  | CFP+all imaging | 4 | (5%) | 28 | (38%) | *34* | *(46%)* | 3 | (4%) | *69* | *(93%)* | 5 | (7%) | 1 | (1%) | *2* | *(3%)* | *2* | *(3%)* |
| Case 5 | CFP only | 11 | (16%) | *20* | *(29%)* | 9 | (13%) | 0 | (0%) | *40* | *(59%)* | 28 | (41%) | 3 | (4%) | *23* | *(34%)* | 2 | (3%) |
| Intermediate | CFP+1 imaging | 4 | (6%) | *17* | *(25%)* | 9 | (13%) | 0 | (0%) | 30 | (44%) | *38* | *(56%)* | 1 | (1%) | *35* | *(51%)* | 2 | (3%) |
|  | CFP+all imaging | 3 | (4%) | *19* | *(28%)* | 7 | (10%) | 0 | (0%) | 29 | (43%) | *39* | *(57%)* | 1 | (1%) | *36* | *(53%)* | 2 | (3%) |
| Case 6 | CFP only | 9 | (12%) | *24* | *(32%)* | 9 | (12%) | 0 | (0%) | *42* | *(56%)* | 33 | (44%) | 1 | (1%) | *31* | *(41%)* | 1 | (1%) |
| Intermediate | CFP+1 imaging | 8 | (11%) | *26* | *(35%)* | 8 | (11%) | 0 | (0%) | *42* | *(56%)* | 33 | (44%) | 0 | (0%) | *32* | *(43%)* | 1 | (1%) |
|  | CFP+all imaging | 5 | (7%) | *28* | *(37%)* | 8 | (11%) | 0 | (0%) | *41* | *(55%)* | 34 | (45%) | 0 | (0%) | *33* | *(44%)* | 1 | (1%) |
| Case 7 | CFP only | 5 | (6%) | *20* | *(26%)* | 21 | (27%) | 1 | (1%) | *47* | *(61%)* | 30 | (39%) | 0 | (0%) | *27* | *(35%)* | 3 | (4%) |
| Intermediate | CFP+1 imaging | 8 | (10%) | 16 | (21%) | *19* | *(25%)* | 1 | (1%) | *44* | *(57%)* | 33 | (43%) | 0 | (0%) | *30* | *(39%)* | 3 | (4%) |
|  | CFP+all imaging | 7 | (9%) | 16 | (21%) | *19* | *(25%)* | 1 | (1%) | *43* | *(56%)* | 34 | (44%) | 1 | (1%) | *31* | *(40%)* | 2 | (3%) |
| Case 8 | CFP only | 8 | (11%) | *21* | *(30%)* | 8 | (11%) | 0 | (0%) | *37* | *(53%)* | 33 | (47%) | 1 | (1%) | *30* | *(43%)* | 2 | (3%) |
| Intermediate | CFP+1 imaging | 5 | (7%) | *20* | *(29%)* | 10 | (14%) | 0 | (0%) | *35* | *(50%)* | *35* | *(50%)* | 0 | (0%) | *33* | *(47%)* | 2 | (3%) |
|  | CFP+all imaging | 5 | (7%) | *19* | *(27%)* | 10 | (14%) | 0 | (0%) | 34 | (49%) | *36* | *(51%)* | 0 | (0%) | *34* | *(49%)* | 2 | (3%) |
| Case 9 | CFP only | *12* | *(16%)* | 7 | (9%) | 3 | (4%) | 0 | (0%) | 22 | (29%) | *54* | *(71%)* | 0 | (0%) | *53* | *(70%)* | 1 | (1%) |
| Advanced | CFP+1 imaging | *4* | *(5%)* | 2 | (3%) | *4* | *(5%)* | 0 | (0%) | 10 | (13%) | *66* | *(87%)* | 0 | (0%) | *64* | *(84%)* | 2 | (3%) |
| GA | CFP+all imaging | *4* | *(5%)* | 1 | (1%) | *4* | *(5%)* | 0 | (0%) | 9 | (12%) | *67* | *(88%)* | 0 | (0%) | *65* | *(86%)* | 2 | (3%) |
| Case 10 | CFP only | *11* | *(15%)* | 7 | (10%) | 5 | (7%) | 0 | (0%) | 23 | (32%) | *50* | *(68%)* | 0 | (0%) | *49* | *(67%)* | 1 | (1%) |
| Advanced | CFP+1 imaging | *10* | *(14%)* | 4 | (5%) | 4 | (5%) | 0 | (0%) | 18 | (25%) | *55* | *(75%)* | 0 | (0%) | *55* | *(75%)* | 0 | (0%) |
| CNV | CFP+all imaging | *8* | *(11%)* | 1 | (1%) | 3 | (4%) | 0 | (0%) | 12 | (16%) | *61* | *(84%)* | 0 | (0%) | *60* | *(82%)* | 1 | (1%) |
|  |  |  |  |  |  |  |  |  |  | 418 | (57%) | 310 | (43%) |  |  |  |  |  |  |
|  |  |  |  |  |  |  |  |  |  | 390 | (54%) | 338 | (46%) |  |  |  |  |  |  |
|  |  |  |  |  |  |  |  |  |  | 383 | (53%) | 345 | (47%) |  |  |  |  |  |  |

Abbreviations: GA, geographic atrophy; CNV, choroidal neovascularisation
